# Translating Combined Mother-Baby Interaction Therapy and Interpersonal Psychotherapy for Postpartum Depression from In-Person to Digital Delivery via *MommaConnect*

**DOI:** 10.18103/mra.v14i2.7298

**Published:** 2026-02

**Authors:** June A. Horowitz, Bobbie Posmontier, Pamela A. Geller, Mary McDonough, Kayla Alvares, Yiqi Wang, Mona Elgohail, Katie Chang, Tony Ma

**Affiliations:** 1 University of Massachusetts Dartmouth, Dartmouth MA U.S.A; 2 Hunter College, New York, NY, U.S.A; 3 Drexel University, Philadelphia, PA, U.S.A; 4 Northeastern University, Boston, MA, U.S.A; 5 Benten Technologies, Manassas, VA, U.S.A

## Abstract

**Background and Purpose:**

Postpartum depression (PPD) is a common childbirth complication that impairs the mother-infant relationship and contributes to long-term developmental challenges among children. Although PPD treatment reduces depressive symptoms, it is not necessarily sufficient to improve maternal-infant interaction. In addition, several barriers preclude timely access to care including mental health stigma, limited specialized providers, and challenges with navigating the healthcare system and securing necessary childcare and transportation. Our team developed a 12-session patient- and clinician-facing digital platform, *MommaConnect* that combines evidence-based therapies-- Mother Baby Interaction Therapy and Interpersonal Psychotherapy-- to improve PPD symptoms and mother-infant interaction, address barriers to care, and provide support between therapy sessions. The purpose of this article is to describe the translation of our in-person approach to treating PPD and improving mother-baby interaction to our *MommaConnect* digital platform that integrates core psychotherapy components into a user-centered platform.

**Methods:**

Our methods were guided by the PRECEDE-PROCEED Model and three stages of a nine-stage digital platform development process. In stage 1, we conducted focus groups to understand the needs of postpartum mothers who had experienced PPD and in-depth interviews to understand the concerns of perinatal mental health clinicians. We analyzed the qualitative data with thematic content analysis. In stage 2, we used component mapping to translate treatment modalities into the digital platform. Finally in stage 3, we used co-design, prototyping, and user-interface design to build the *MommaConnect* prototype.

**Results:**

Results from the focus groups identified four themes including (1) barriers to accessing care, (2) preferences and needs in mental health care delivery, (3) feedback on the *MommaConnect* App, and (4) broader challenges and experiences. The in-depth interviews with the clinicians resulted in six themes including (1) assessment and early intervention, (2) barriers to care, (3) role of technology in treatment, (4) building a therapeutic alliance, (5) community and social support, and (6) addressing comorbid conditions. Additionally, the team translated the in-person treatment modality to a 12-session format and designed the current version of the *MommaConnect* prototype, which has both patient-facing and clinician-facing components.

**Conclusions and Implications:**

*MommaConnect* offers an innovative, accessible approach that combines Mother Baby Interaction Therapy and Interpersonal Psychotherapy to treat PPD and improve the quality of mother-infant interaction. Its digital adaptation provides a treatment platform for mothers and clinicians to work together in real time and communicate between sessions. *MommaConnect* may expand access, reduce barriers to care, and inform the broader translation of evidence-based behavioral interventions into scalable, technology-enabled formats. This work has the potential to transform access to perinatal mental health care, improve maternal and infant outcomes, and reduce the long-term sequelae associated with untreated PPD and disrupted early relational experiences for infants.

## Introduction

Postpartum depression (PPD) is a common childbirth complication that impairs maternal functioning and disrupts the developing mother–infant relationship.^1–3^ Mothers with PPD may experience a variety of symptoms including persistent sadness, reduced pleasure, poor concentration, anxiety, irritability, sleep disruption, and psychomotor agitation or delayed reactions.^4,5^ Sadly, many women face persistent barriers that limit access to timely, evidence-based, in-person care for PPD.^6,7^ Beyond the significant consequences on maternal health resulting from their own symptoms and possible delay in care, PPD can also lead to impaired responsiveness to the infant and decision making, thereby affecting the quality of mother-infant interaction.^8^

The interconnectedness of PPD symptoms and quality of mother-infant engagement creates a harmful cycle in which relationship difficulties can worsen depressive symptoms. Research by Beck indicated that maternal PPD substantially disrupts mother–infant interaction in the first year of life with moderate-to-large negative effects.^9^ This disrupted interaction creates a harmful feedback loop for the dyad. Difficulty with how to respond appropriately to their infants can result in infant distress, increased crying, and feeding difficulties. This then triggers feelings of maternal inadequacy, guilt, and failure, which further worsen depressive symptoms.^8^ The opposite is also true with positive feedback where mothers with strong maternal-fetal/infant attachment demonstrate higher maternal sensitivity and ability to analyze and respond to infant cues, providing some protection against the deterioration cycle.^10,11^ Therefore, treating maternal depression alone is necessary but insufficient to repair early relational disruptions. As such, scalable interventions that address both maternal depressive symptoms and mother–infant interaction are urgently needed.^2,7,12^

To meet this need, we are developing a novel digital platform, *MommaConnect*, that integrates both clinician- and patient-facing features, psychoeducation, and therapeutic sessions grounded in combined Mother Baby Interaction therapy (MBI) and Interpersonal Psychotherapy (IPT) which we call *MBIPT*. Based on our experience delivering treatment in our in-person intensive perinatal outpatient program (*Mother Baby Connections*)^13^ and guided by a multi-method, user-centered development process, we first sought to understand mothers’ needs through focus groups and explore clinical priorities through in-depth interviews with frontline clinicians. Using this feedback, we then translated core components of in-person therapy using component mapping and expert panel review to compile the digital platform prototype in collaboration with digital developers.

Thus, the purpose of this article is to describe the translation of the *Mother Baby Connections* approach from an in-person intervention for maternal depression and mother-infant interaction to a more accessible, digitally enhanced approach, *i.e., MommaConnect*, an application that integrates critical treatment components into a user-friendly platform, with clinician- and patient-facing tools available during and between sessions.

## Background and Rationale

Within the first year postpartum approximately one in seven women experience PPD, with higher rates among subgroups including African American and Black mothers.^1,2,14^ PPD as discussed above is marked by symptoms that negatively impact both maternal well-being and the quality of mother-infant interactions.^4,5,15–17^ When left untreated, PPD initiates a cascade of interconnected problems affecting the mother, her family and the infant.^18^ In addition to diminishing quality of maternal-infant interaction, maternal depression can contribute to strained relationships with partners and family and disrupt caregiving—factors that increase infant stress reactivity and in turn exacerbate maternal PPD symptoms and risk for chronic depression.^15–19^

Untreated PPD significantly harms the developing mother-infant bond, with downstream consequences for the infant’s growth and development.^11,16,17,20,21^ Mothers with PPD often show decreased responsiveness, lower positive affect, reduced sensitivity, and less engagement with their babies during caregiving,^22^ creating a harmful cycle amplifying maternal distress and worsening infant socio-emotional and cognitive development.^8,9^ Children of mothers with untreated PPD face increased risk for developmental delays, behavioral and attention difficulties, depression, anxiety, and impaired emotional regulation that can extend well into adulthood.^8,23–26^ Moreover, research findings have linked PPD with insecure maternal-infant attachment that can persist long after PPD symptoms have resolved.^10,11^

Unfortunately, up to 80% of women with PPD fail to receive appropriate treatment, creating a profound gap in care that drives persistent mental health disparities and leaves many mothers without needed support.2 Although existing evidence-based treatments can effectively reduce maternal depressive symptoms, they often fail to address the maternal-infant relational difficulties central to PPD. Standard interventions such as antidepressant medications and individual psychotherapy typically improve depressive symptoms but do not directly improve impaired mother-infant interaction.^12^ Additionally, such traditional treatments often require several months to produce noticeable clinical improvement, during which the quality of mother-infant interaction may remain compromised.^12^ Given the rapid infant development during the postpartum period, delays in addressing maternal-infant interactions represent missed opportunities for fostering secure attachment and healthy infant development.^8,23–26^ Effective interventions must therefore address maternal depressive symptoms *and* dyadic functioning to break this cycle and safeguard maternal mental health, the dyadic relationship and child well-being.^3,27^

### Combined IPT and MBI Treatment Approach.

Within our *Mother Baby Connections* program, we offer a combination of evidence-based individual, dyadic and group treatments including IPT to treat PPD symptoms combined with MBI to address the maternal-infant relationship.

Interpersonal Psychotherapy is a time-limited treatment used for more than 40 years to treat women experiencing PPD.^28,29^ In traditional IPT, the focus of therapy is on adult relationships and the social context of depression, including interpersonal role disputes, role transition difficulties, social isolation and/or grief. The premise of IPT is to link depressed mood with interpersonal problems and improve relationships and expectations with partners, family, and wider social networks. During the initial sessions, the therapist establishes the diagnosis of PPD and then works with the mother to explore her social functioning, close relationships, communication patterns, interpersonal expectations and the social context in which depressive symptoms emerged. Once problems are identified, the therapist works with the mother during the middle sessions to analyze her communication patterns and uses decision analysis and behavioral changes to resolve interpersonal dysfunction. Women with PPD learn to communicate their personal needs more effectively to others and to engage their social networks for support. During the final sessions, the therapist and mother review her newly learned interpersonal skills, and the therapist reinforces her competence and plans for therapy termination. Mother-Baby Interaction Therapy promotes responsive interaction between mothers’ experiencing PPD and their infants,^13,16,17,20^ The role of the clinician is to support the mother to optimize the quality of her interaction with her baby by: 1) teaching her about engagement and disengagement infant cues and 2) directly coaching her to shape maternal responsive behaviors via modeling and giving feedback with behavioral shaping, support, and praise for contingent responsive maternal behaviors when displayed.

Mother-baby Interaction Therapy is tailored to the individual mother’s interactive repertoire and the infant’s cues and age/developmental status. For example, when a mother shows limited or delayed responsiveness to her infant’s engagement cues, the clinician models contingent responsive actions and identifies such responses by the mother as they occur, such as reinforcing and coaching responses when her baby shows readiness by talking, touching, and/or using motion to engage. In contrast, when a mother displays intrusive interactions, the clinician encourages the mother to adjust her responses to match the baby’s timing and developmental stage. Thus, suggestions for interacting with the infant are tailored to the infant’s cues, age, and development. Positive feedback and support are interlaced throughout the behavioral coaching process.

### Treatment Barriers.

Despite effective treatments, interconnected barriers to accessing comprehensive perinatal mental health care persist. System-level limitations include a severe shortage of specialized services. For example, at present, only 45 perinatal intensive outpatient mental health programs, and five specialized perinatal mental health inpatient units exist in the United States.^30^ Moreover, only seven states have even 1% of psychiatrists specializing in perinatal mental health, highlighting an extremely limited national workforce capacity.^22,31^ In addition, 61% of rural counties lack any psychiatric provider whatsoever, further restricting access to mental health care for perinatal populations.^22,31^

At the individual level, mental health stigma, limited awareness of maternal mental health conditions, and failure to recognize depressive symptoms impede treatment-seeking.^2^ Practical challenges further restrict access. Childcare responsibilities are a major barrier, as many mothers cannot attend appointments without bringing their children, and alternative childcare arrangements may be unavailable or unaffordable.^6^ Work schedule conflicts and the unpredictable nature of infant care needs exacerbate these obstacles.^6^ Collectively, these practical barriers contribute to high no-show rates^7^ reinforcing the cycle of untreated depression and further disrupting the mother-infant relationship. Strategies to bridge these barriers are essential.

### Digital Solutions.

Our review of market research on existing iOS and Android applications for PPD identified critical gaps in digital interventions to integrate therapeutic components with mother-infant interaction support. Digital delivery expands access to evidence-based PPD care, thereby mitigating the barriers and critical gaps that cause most women who screen positively for depressive symptoms to receive no care.^32^ In addition, telehealth removes geographic, transportation, and childcare barriers by bringing care into the home and offering flexible scheduling that fits infant routines and work demands.^6^ In mental health and perinatal settings, telehealth achieves mental health outcomes comparable to in-person care and reduces missed appointments.^33,34^ Mobile applications complement telehealth with 24/7 psychoeducation, mood and sleep tracking, reminders, and secure messaging, supporting continuous engagement and timely clinical responses with reported satisfaction ranging from 86% to 95%.^35,36^ Digital platforms also allow discreet screening and education that can lessen stigma and encourage help-seeking. Taken together, telehealth plus an integrated mobile application have strong potential to help overcome transportation, childcare, scheduling, geographic, and stigma-related barriers, creating more equitable access to evidence-based PPD treatment regardless of a mother’s location or resources.

#### Momma Connect.

In response to these challenges, our team developed *MommaConnect* that aims to fill existing treatment gaps by adapting treatments for maternal depression and impaired mother-infant interaction from our in-person *Mother Baby Connections* program^13^ into an accessible digital platform combined with telehealth that includes patient- and clinician-facing components. This integrated model provides accessible, relationship-centered care that addresses both maternal depressive symptoms and quality of mother-infant interaction simultaneously during the critical early postpartum window. Through its dual focus on individual maternal mental health and dyadic relationship functioning, *MommaConnect* is designed to disrupt the cycle of maternal depression and impaired parenting, supporting optimal outcomes for both mothers and their infants during this vulnerable developmental period.

## Methods

We guided translation of our face-to-face treatment approach used at *Mother Baby Connection* to the online *MommaConnect* digital healthcare platform with the PRECEDE-PROCEED Model, a planning framework that incorporates epidemiological, environmental, behavioral, and social factors to design health promotion programs.^37^ In addition, we are using a nine-stage process to standardize *MBIPT* session content and translate it for digital delivery. The nine stages include understanding needs; translating therapy; designing the platform; testing usability/feasibility, and efficacy; providing clinical validation; deploying; monitoring development; and ensuring attention to ethics and safety (see Table 1). In this paper, we describe stages 1–3 for development of *MommaConnect*.

### Stage 1 Understanding Needs.

For our initial formative research, we conducted focus groups with women in the perinatal period and in-depth interviews with mental health clinicians to understand the needs, preferences and obstacles relevant to mental health treatment for mothers with PPD, and garner input to inform early digital platform development and co-creation of the *MommaConnect* prototype. All focus groups and interviews were audio-recorded, transcribed verbatim, and analyzed using thematic content analysis.

#### FOCUS GROUPS.

To guide our work, our initial focus groups were comprised of six women from the general perinatal population. Inclusion criteria were having delivered a baby within the past 12 months, being aged 18 to 44 years, owning a smart phone and having a singleton infant up to 12 months old, and living with the infant. In recognition of higher prevalence of PPD among African American and Black (AA/B) women, we later interviewed six AA/B postpartum mothers in focus groups to co-create the *MommaConnect* prototype. Inclusion criteria were identification as AA/B, being aged 18 to 44 years, owning a smart phone and having a singleton infant up to 12 months old, living with the infant, and self-reported PPD.

We sought to understand treatment needs and barriers to care for this population, and to include those findings in the design development. We conducted three focus groups using a moderator guide. Specifically, we asked about mental health perceptions, cultural and socioeconomic facilitators and barriers to care to solicit a conversation and understanding of their perspectives. We showed mothers early draft mock-ups of the *MommaConnect* prototype and asked targeted questions about design and functionality including onboarding, homepage layout, daily tracking features (e.g., mood, sleep), between-session activities, mental health support, psychoeducation on PPD and maternal-infant interaction, frequently asked questions (FAQs), the resource library, links to social media, and the emergency support referral page.

#### IN-DEPTH INTERVIEWS.

In parallel with focus groups, we conducted in-depth interviews with mental health clinicians. To inform *MommaConnect’s* clinician-facing features, we recruited eight providers (i.e., social workers, psychologists, psychiatric nurse practitioners, and a psychiatrist) who met the inclusion criteria (i.e., over the age of 18, English speaking, had at least 1 year of experience providing mental health care to the postpartum population, and had patients who were AA/B). We sought to identify features that would support clinical documentation, address perceived patient barriers to care, facilitate troubleshooting, align with clinician preferences for telehealth delivery, and streamline processes for client intake and ongoing management of postpartum mental health disorders. Just as with the focus groups, we used an interview guide for consistency.

##### DATA ANALYSIS.

We conducted content analysis^50^ of transcripts from the focus groups and in-depth interviews to identify and extract themes from the qualitative data. Following established qualitative research methodology, two investigators independently reviewed all transcripts and engaged in open coding to identify initial codes. Codes were then organized into broader categories and themes. The investigators met regularly to discuss emerging patterns, resolve discrepancies through discussion, and reach consensus on the final thematic structure. These themes directly informed the refinement of both the patient-facing and clinician-facing components of the *MommaConnect* platform. We analyzed demographic data using descriptive statistics.

### Stage 2 Translating Therapy.

Based on the findings from the focus groups and the in-depth interviews and our experience with in-person treatment delivery through Mother Baby Connections^(e.g., 13)^, we used component mapping and expert clinicians on our research team to determine the general framework and session format and develop the specific content for 12 therapeutic sessions grounded in combined Mother Baby Interaction therapy and Interpersonal Psychotherapy (MBIPT).^13,51–53^ We also created a psychoeducation library to be used with our patient- and clinician-facing digital approaches.

We first used component mapping to organize MBI and IPT components into structured session content for the combined MBIPT intervention. We mapped the core components from each modality to specific session activities based on their targeted processes, allowing for integrated delivery of interpersonal and dyadic therapeutic procedures within a unified treatment framework. Specifically, the IPT components addressing interpersonal stressors, role transitions, affect regulation, and social support were mapped to session activities emphasizing maternal emotional experience and relational context, while MBI components targeting maternal sensitivity, responsiveness to infant cues, and dyadic attunement were mapped to sessions incorporating interaction-focused procedures and activities (e.g., observation of mothers’ videos of their interaction with their infants). Shared processes, such as emotional attunement and reflective functioning, were intentionally integrated within sessions. After the content for each session was refined and standardized, our team collaborated to translate the material into a digital format suitable for clinician- and patient-facing delivery.

**Figure 1.**
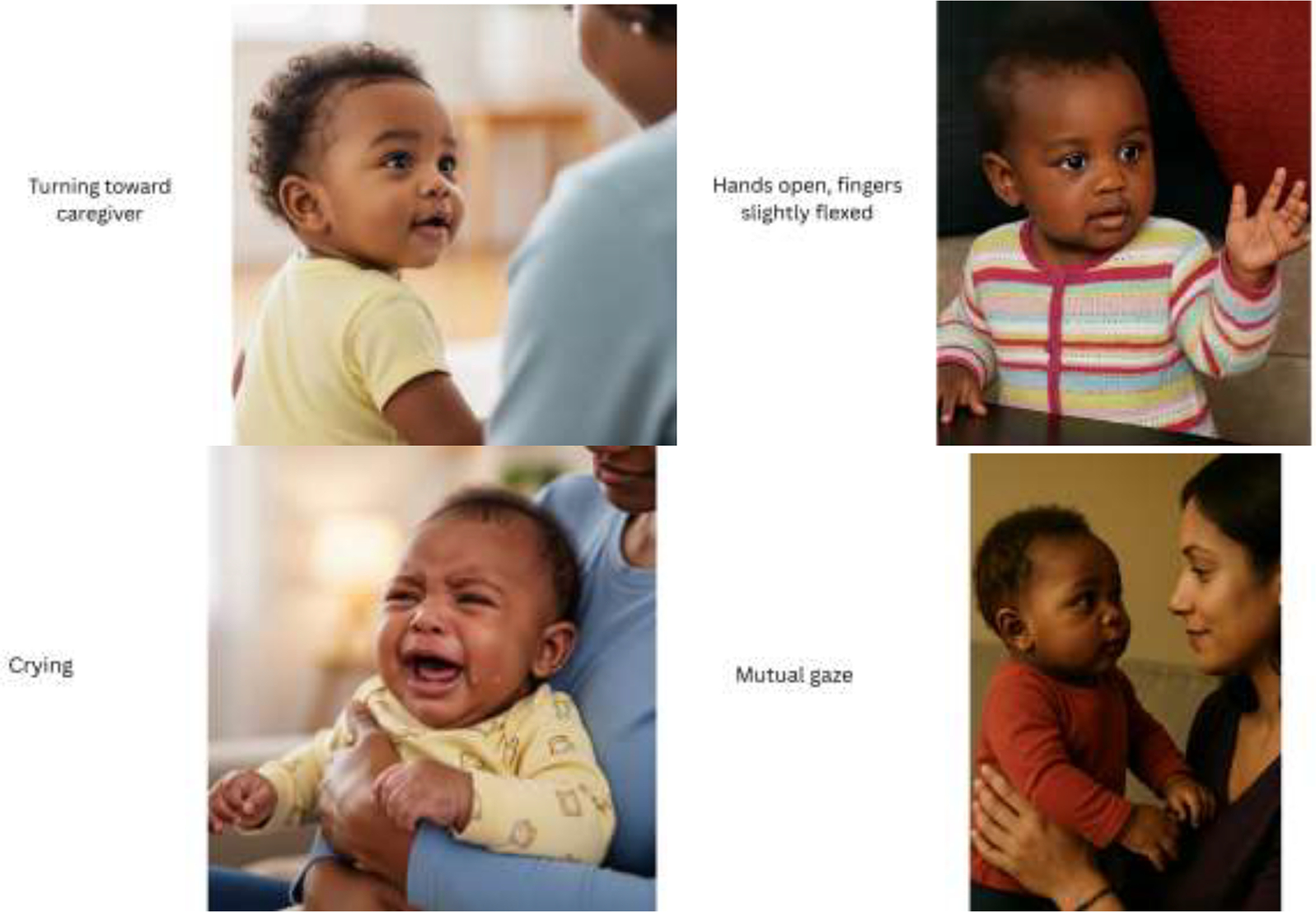
Examples of baby cues images. Note: Examples of baby cues featuring African American/Black infants, developed using AI-assisted image generation and validated by clinical experts. Cues depicted include engagement signals (e.g., turning toward caregiver, hands open with fingers slightly flexed, mutual gaze) and disengagement signals (e.g., crying).

Our team also developed visual aids to teach mothers about baby cues as an essential component of MBIPT. We systematically created images to display baby cues to promote mothers’ abilities to recognize and interpret their infants’ behaviors. (Figure 1) We used the *Nursing Child Assessment Satellite Training* (NCAST) *BabyCues* materials (2014 Parent-Child Relationship Programs University of Washington Seattle. WA),^54^ as our “gold standard” for illustrating baby engagement and disengagement cues by identifying essential behavioral indicators across cue categories. Using AI generation, we created multiple versions depicting AA/B infants across ages (newborn-12 months), settings (feeding, play, sleep), and emotional states (content, distressed, engaged, disengaged). We assessed each image for accuracy, cultural authenticity, clarity, and emotional resonance, requiring multiple iterations for quality assurance. To reach consensus among our expert panel, we altered images that appeared robotic or did not match baby cue characteristics. We also facilitated mothers’ ability to video-record brief mother-baby interactions and upload directly to *MommaConnect* to be viewed by the clinician for feedback and coaching and to evaluate the quality of maternal-infant interaction.

### Stage 3 Designing the platform.

By collaborating with user interface/user experience (UI/UX) designers, we created initial low-fidelity wireframes outlining navigation, key features (i.e., session scheduling, educational libraries, maternal-infant interaction video upload for interaction review/coding, mood tracking, and therapist messaging), and user flow. Modified wireframes based on mothers’ feedback emphasized streamlined navigation and representative imagery. We then developed high-fidelity clickable wireframes with finalized color schemes and culturally tailored design informed by mothers’ feedback. We conducted backend database development to support secure, Health Insurance Portability and Accountability Act (HIPAA)-compliant data storage and communication. We researched and drafted content sheets for daily affirmation quotes and frequently asked questions (FAQ) sections, ensuring cultural appropriateness and health literacy, accessibility, and planned micro-learning videos covering postpartum depression, maternal-adult/maternal-infant interpersonal relationships with extended IPT, baby cues tailored for African American/Black infants with AI-assisted development and expert validation, and mother-infant interaction.

## Results

### Stage 1

Findings from maternal focus groups and clinician in-depth interviews revealed critical insights into treatment targets, systemic barriers, cultural considerations, and technology preferences that directly shaped the design and functionality of *MommaConnect* that included: (1) barriers to accessing care, (2) preferences and needs in mental health care delivery, (3) feedback on the *MommaConnect* App, and (4) broader challenges and experiences. The in-depth interviews revealed six themes including (1) assessment and early intervention, (2) barriers to care, (3) role of technology in treatment, (4) building a therapeutic alliance, (5) community and social support, and (6) addressing comorbid conditions.

## Focus Groups Themes

### 1. Barriers to Accessing Care

This theme reflects barriers identified by mothers that inhibit their ability to access care. Three subthemes emerged: limited support systems for African American mothers, insurance and financial barriers to mental health care, and difficulty finding culturally competent providers.

*1)Limited support systems for African American mothers*. Mothers identified a lack of community support as a significant barrier to accessing care. One mother stated, “Nobody listens to us, even in medical settings” (FGP3) highlighting how easily AA/B women can be dismissed within the healthcare system. Participants also emphasized the importance of provider representation, expressing a desire for clinicians “from different backgrounds and races; diverse doctors” (FGP4) as a means of reducing barriers to care.

*2)Insurance and financial barriers to accessing mental health care*. Mothers identified how insurance coverage and “financial constraints, despite telehealth options” (FGP3) impede their ability to access mental health services. These financial burdens often deter mothers from seeking the care they need.

*3)Difficulty finding culturally competent providers*. Mothers expressed a desire for providers who understand their lived experiences and unique perspectives. One mother stated, “healthcare providers need to understand black women’s needs” (FGP4) underscoring the importance of provider representation and cultural humility in reducing barriers to care.

### 2. Preferences and needs in mental health care delivery

Mothers identified what they were looking for in terms of preferences for healthcare delivery, especially within a digital platform. Three subthemes emerged including: preference for telehealth/remote delivery, importance of self-care activities, desire for peer support and community connection.

*1)Preference for telehealth/remote delivery*. Mothers were receptive to treatment on a digital platform stating, “telehealth is incredible, avoiding travel with a baby” (FGP3) and that it is “more convenient and comfortable than in-person visits” (FGP4).

*2)Importance of self-care activities*. Mothers emphasized the importance of self-care activities in digital health interventions, alongside formal treatment. Mothers expressed a desire for support and encouragement for self-care activities such as “nature walks, writing poetry, and reading books” (FGP4).

*3)Desire for peer support and community connection*. Another feature mothers expressed interest in was the ability to connect with the community and receive support from others in similar situations. Mothers sought an opportunity to connect with other mothers noting they “would love a play day; spending time with other kids and parents” (FGP3) to increase their social engagement. They also expressed that they “love support groups; [they’re] fun to join and talk to people” (FGP3). This desire to connect with peers highlighted the value mothers placed on peer connection and social support.

### 3. Feedback on *MommaConnect* app

Mothers provided feedback on the *MommaConnect* app that highlighted both strengths of existing features and suggestions for enhancements to improve the platform.

#### Positive reception.

Overall, the app was well received. Mothers described *MommaConnect* as a “good idea,” and noted its delivery would make it “comfortable for Black women to join” (FGP4). Mothers highlighted the app’s simplicity and ease of use as key strengths. Lastly, mothers appreciated the ability to track sleep for both them and their infants.

#### Suggestions for app improvement.

Mothers offered feedback to enhance the app’s usability and relevance. Suggestions included options to increase personalization, such as allowing users to select a “different color palette” (FGP3) and the ability to switch language settings so the app could be used in their native language, if not English. Mothers also emphasized the importance of being able to “enhance privacy settings” such as including “Face ID for iPhones” (FGP4). Key motivators for engagement included addressing the loneliness of motherhood and connecting with other mothers who share similar experiences. Mothers expressed interest in additional features such as progress tracking, easy appointment access, diverse imagery, assignment checklists, emergency support resources, journaling capability, and personalization options.

### 4. Broader challenges and experiences

Mothers discussed broader challenges and experiences related to PPD as AA/B women navigating healthcare. Three subthemes emerged including challenges with family dynamics, challenges in navigating the healthcare system, and impact of postpartum depression on daily life.

*1)Challenges with family dynamics*. Challenges with family relationships impacted how mothers rely on them as sources of support. Mothers described “complicated relationships” including family members who “don’t respect boundaries” (FGP3). Others noted limited communication with family stating they “don’t really talk to family” (FGP4) further constraining family support.

*2)Challenges in navigating the healthcare system*. Mothers described significant difficulty navigating the healthcare system during the postpartum period while managing PPD symptoms. One mother highlighted how difficult it is “to get to appointments after having a baby” citing physical and emotional changes, finding childcare and the necessity of bringing the infant to visits (FGP2). Mothers also reported feeling “overwhelmed with tasks” (FGP2) when scheduling appointments and coordinating care.

*3)Impact of postpartum depression on daily life*. PPD substantially affected mothers’ daily functioning. They described feeling overwhelmed by everyday responsibilities, but the added burden of “severe postpartum depression” created further barriers to daily life (FGP1). One mother described PPD as a “very scary experience with my first child” (FGP1), illustrating the layered emotional and functional challenges during the postpartum period.

#### In-depth Interview Themes

The in-depth interviews with perinatal mental health clinicians provided insight into strategies to provide optimal support for African American/Black women with PPD, as well as key components to incorporate into the clinician-facing part of the digital platform. Six themes were identified including: (1) assessment and early intervention, (2) barriers to care, (3) role of technology in treatment, (4) building a therapeutic alliance, (5) community and social support, and (6) addressing comorbid conditions.

##### 1. Assessment and Early intervention

The clinicians expressed the importance of assessment and early intervention among mothers at risk for PPD, and communicating findings with their obstetricians. This close monitoring ensured “that the support systems are in place to watch for symptoms” (IDIC6). The importance of following up and providing ongoing support “means bringing them back in for their visits sooner rather than later” (IDIC6), to allow for better management of symptoms.

##### 2. Barriers to care

Barriers to care identified by clinicians included insurance and cultural factors. One clinician stated that “one of the major systemic barriers is insurance-based” (IDIC6). However, when insurance was accepted patients “weren’t able to specifically [find] somebody by race” to match their own experiences (IDIC2). Other barriers to care included culture and stigma. One clinician noted that “some cultures are taught a lot of resilience…African Americans tend to be very resilient, I think, or minimizing symptoms of postpartum depression” (IDIC6). In general, “there’s a stigma about mental illness in the African American community” and “[many] believe they can pray…mental illness into wellness” (IDIC6).

##### 3. Role of technology in treatment

Findings indicated that technology can be a useful tool for accessing care by reducing disruptions to patients’ daily lives. The use of technology treatment “doesn’t remove [mothers] from [their] routine” (IDIC6). One clinician described telehealth as “absolutely incredible” as patients no longer need to worry about getting “everything together enough to get out of the house with a baby” to receive treatment (IDIC3). Clinicians also suggested that extending therapeutic contact beyond synchronous sessions through mobile application features could help mothers stay engaged with treatment and provide between-session support and psychoeducation.

##### 4. Building a therapeutic alliance

An important part of treatment is building a therapeutic alliance and providing culturally competent care. One clinician recommended “engaging [mothers] with things that they would want to talk about, being personal” (IDIC6). Another clinician stated how representing the community they are treating is “helpful because people can identify with it” which is important to reaching the African American/Black community (IDIC6).

##### 5. Community and social support

Clinicians offered several recommendations for education to support African American/Black postpartum women. Community-based education was viewed as a key strategy for reducing stigma and creating space for “women to talk about postpartum depression” (IDIC6). Enhancing this sense of community for women may help reduce feelings of isolation among postpartum women.

Clinicians also emphasized the importance of affirming mothers’ and personhood, reinforcing that they “are still a whole person on [their] own” and are “allowed to enjoy things for [themselves], to take self-care” (IDIC6). Such support acknowledges and addresses the multiple identities and roles women navigate during the postpartum period.

##### 6. Addressing comorbid conditions

Finally, clinicians identified the need to understand and address comorbid conditions to tailor treatment effectively for women with PPD. They highlighted the impact of co-occurring symptoms such as anxiety, obsessive compulsive disorder, and post-traumatic stress disorder on the presentation and course of PPD (IDIC5). Clinicians also noted that prior interventions for these conditions may have resulted in negative experiences with mental health care, which can influence patients’ engagement in subsequent treatment.

#### Stage 2 Translating Therapy

As with our in-person treatment, we extended the traditional IPT focus on adult interpersonal relationships to emphasize the mother–infant dyad and the broader social network that supports both partners. Our clinical MBIPT session content highlights the bidirectional influence of maternal mood on the developing infant relationship and the ways infant behaviors can, in turn, affect maternal emotional regulation and stress. IPT activities emphasize the mother’s ability to evaluate and mobilize a supportive social network, and construct a comprehensive interpersonal inventory and biopsychosocial formulation tailored to perinatal needs.

Key therapeutic processes of our MBIPT approach include identifying interpersonal problem areas, recognizing and validating affect, and learning communication analysis and decision analysis strategies relevant to both adult relationships and mother–infant interaction patterns. This dyad-focused content is designed to strengthen maternal emotional attunement, improve relational communication, and address disruptions in caregiving and bonding.

Our research team created the framework for the 12-session MBIPT platform including setting the agenda, mindfulness exercises, general check-ins, session components and between-session activities. In addition, the sessions include measures for improving maternal depression symptom severity, infant and maternal sleep, mother-infant attachment and mother-infant interaction via review of videos by the clinician with the mother. Among the 12 sessions, the first focused on an introduction to the digital platform and reviewing emotions, and the last module focused on relapse prevention and establishing follow up care if needed beyond the timed intervention. Among the remaining sessions, four focused on mother-infant interaction, four focused on adult interpersonal relationships and support, and two on a combination of adult relationships and the relationship with the infant. This framework was shared with the digital developers on the team to translate the content into digital delivery.

#### Stage 3 Designing the platform

The translation process resulted in a comprehensive prototype of our digital health platform. The *MommaConnect* architecture was designed around five core navigation sections (i.e., Home, Library, My Progress, Support, and Profile) enabling mothers to access different features based on their immediate needs. In the following sections, we highlight key features of the *MommaConnect* design.

Therapeutic Delivery and Communication Features. *MommaConnect* facilitates therapeutic engagement through both synchronous and asynchronous modalities to address the flexibility and safety needs identified in stakeholder feedback. Mothers connect with their assigned therapists through scheduled video sessions for real-time therapy delivery and secure text-based messaging for communication between appointments. This multimodal approach allows mothers to choose the communication method that best fits their immediate circumstances, including switching to text-based communication when safety or privacy concerns arise due to the presence of partners or others in the home environment.

Structured Activities and Self-Monitoring Tools. The Home dashboard provides personalized weekly therapeutic activities organized by treatment sessions. Mothers can engage in self-care, view psychoeducational videos, and complete depression and mother-infant attachment questionnaires. The platform enables mothers to monitor their wellbeing across multiple dimensions through integrated check-in features for mood, sleep, journaling, and self-care activities. These tools use intuitive interfaces that minimize user burden while supporting ongoing symptom awareness. Progress charts visualize mood and symptom patterns over time, allowing both mothers and clinicians to track changes and evaluate treatment.

Psychoeducational Content Library. The library offers a comprehensive collection of educational materials organized by relevant topics, including general depression and PPD, anxiety and postpartum anxiety, emotional wellbeing, stress management, relaxation techniques, mindful eating and drinking, and mother-baby interaction. Content is available in multiple formats, including educational videos and audio podcasts. The library includes search and bookmarking capabilities so mothers can easily find and save materials relevant to their needs.

Progress Tracking and Engagement Features. The My Progress section provides visual feedback on treatment advancement through progress indicators showing completed sessions. This feature addresses mothers’ expressed desire for clear markers of accomplishment and progress throughout their therapeutic journey. The personalized home screen welcomes mothers by name and displays daily motivational quotes with optional feedback prompts, creating a more personalized and engaging user experience.

Integrated Support Resources. The Support section brings together multiple layers of assistance in one accessible location. *MommaConnect* provides direct access to crisis support services, including the United States-based National Maternal Mental Health Hotline and National Suicide Prevention Lifeline, and Postpartum Support International resources, with simple one-tap calling functionality.

**Figure 2.**
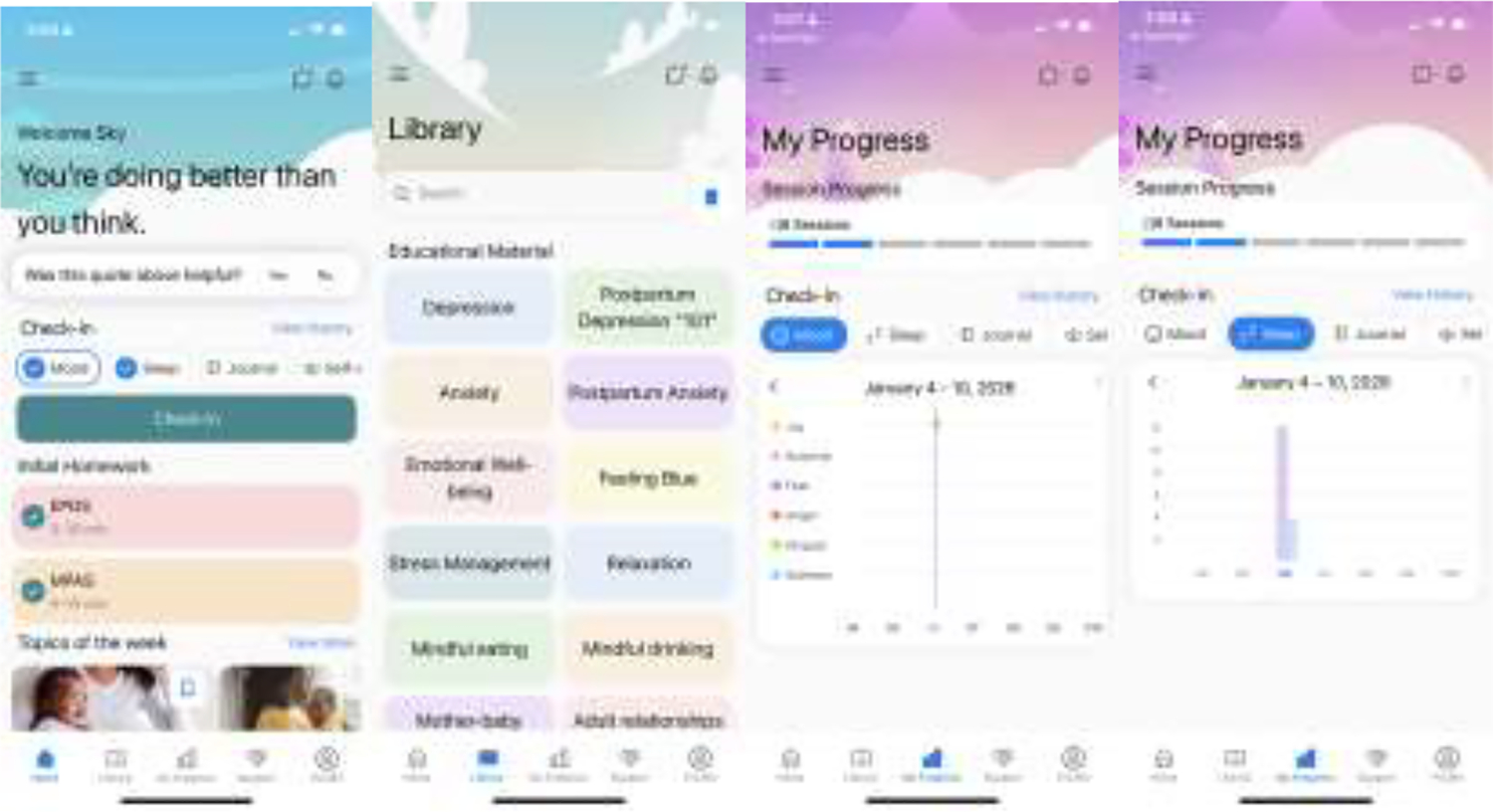
Screenshots of *MommaConnect* mobile application Note: Screenshots of the *MommaConnect* mobile application showing key features: home dashboard with daily checkins and affirmations, psychoeducational resource library, and progress tracking with mood and sleep visualization.

User Interface Design (Figure 2). The platform features a soothing visual design with soft color gradients and diverse imagery of mothers and infants. Clear navigation with recognizable icons and labels makes the platform easy to use, while message and notification features keep mothers connected to their therapists and informed of important updates.

## Discussion

We described the first three stages (understanding needs, translating therapy, and designing the platform) of a nine-stage process of translating Mother Baby Interaction Therapy and Interpersonal Psychotherapy from an in-person behavioral and coaching and psychotherapy intervention to improve maternal depression and mother-infant interaction into a more accessible, digitally augmented clinician- and patient- facing mobile application, *MommaConnect*. This work addresses a critical gap in PPD literature by demonstrating how a relational, dyadic-focused psychotherapy can be systematically adapted into a user-centered digital platform while preserving core therapeutic mechanisms.

Findings from maternal focus groups reflected well-documented barriers that inhibit access to postpartum mental health care, including difficulty finding culturally responsive providers, challenges navigating the healthcare system, and the competing demands of childcare, transportation and daily responsibilities. Mothers also expressed a strong preference for remote treatment that preserves privacy, supports self-care, facilitates peer connection and fosters a sense of community to address isolation and loneliness.^55–57^ Clinician in-depth interviews reinforced these themes by emphasizing the importance of assessment and early intervention, and advantages of continuous availability of asynchronous commination through telehealth, culturally responsive therapeutic alliance building, psychoeducation, and the need to address co-morbid mental health conditions. Together these findings underscore the mismatch between postpartum mothers’ needs and the structure of existing PPD interventions that *MommaConnect* is designed to overcome.

In response to participants’ feedback, we developed *MommaConnect* comprised of 12 sessions. The current platform digitally integrates core components of our in-person mother-baby perinatal mental health program (*Mother Baby Connections*) including Mother Baby Interaction Therapy and Interpersonal Psychotherapy, supplemented with mindfulness exercises, general check-ins, and between-session activities. Unlike many PPD interventions that prioritize symptom reduction alone, *MommaConnect* highlights the bidirectional influence of maternal mood and infant behavior within a scalable digital format.

Participant feedback guided iterative refinement of the platform, resulting in the integration of structured activities, symptom monitoring tools, psychoeducation, progress tracking and engagement features, and support resources. Design enhancements emphasized a user-friendly interface, secure messaging, and notification features to sustain engagement and maintain connection between mothers and therapists. These refinements align with emerging evidence that engagement, usability and therapeutic continuity are critical determinants of effectiveness in digital mental health interventions.^58^ and systemic factors that shape access and engagement.

As with our in-person treatment, we tailored traditional Interpersonal Psychotherapy to emphasize the mother–infant relationship and the broader social network that supports both the mother-infant relationship and other significant relationships. Our tailored version of Interpersonal Psychotherapy complements the behavioral coaching of Mother Baby Interaction Therapy to strengthen maternal emotional attunement, improve relational communication, and address impairments in caregiving and bonding commonly associated with PPD.

Building on this foundation, our next steps for *MommaConnect* include iterative usability and feasibility testing, followed by a randomized controlled trial to establish clinical efficacy. Ultimately, *MommaConnect* aims to fill persistent treatment gaps by translating evidence-based, relationship focused care from in-person delivery to an accessible digitally augmented intervention. *MommaConnect* offers a novel scalable model of dyadic care designed to disrupt the cycle of maternal depression and impaired mother-infant interaction. Our approach aims to support optimal outcomes for both mothers and their infants during this vulnerable developmental period. Future development will be guided by implementation science frameworks and informed by engagement analytics, ecological momentary assessment and ongoing safety monitoring.^59^

## Strengths and Limitations

This work has several notable strengths that contribute to the advancement of digital interventions for PPD. First, *MommaConnect* is grounded in a theory-driven, evidence-based clinical model, integrating Mother Baby Interaction Therapy and Interpersonal Psychotherapy within a digital platform.

Second, the intervention was developed using a user-centered, participatory design process, incorporating perspectives from both postpartum women and perinatal mental health clinicians. The inclusion of both patient- and clinician-facing components further enhances the clinical credibility of *MommaConnect* and differentiates it from fully automated digital self-help approaches that may lack sufficient safety monitoring or therapeutic depth.

Third, *MommaConnect’s* dyadic focus is a significant strength. By addressing maternal depressive symptoms alongside impairments in mother–infant interaction and broader social support, the platform emphasizes interpersonal and developmental processes. The integration of symptom monitoring, psychoeducation, engagement features, and asynchronous clinician communication supports continuity of care while accommodating the practical needs of women during the postpartum period.

Despite these strengths, several limitations warrant consideration. This paper reports only the initial stages of intervention development, and findings are based on qualitative data from maternal focus groups and clinician interviews rather than clinical outcome data. As such, conclusions regarding feasibility, acceptability, and efficacy must be considered preliminary until confirmed through systematic usability/feasibility testing and clinical trials.

Additionally, although participants highlighted the importance of culturally responsive care, the extent to which the current prototype fully addresses the diverse needs of postpartum populations requires further evaluation. Future work should include additional development and broader testing across diverse groups to assess generalizability and inform additional cultural tailoring.

Taken together, these strengths and limitations underscore the importance of continued iterative development and empirical evaluation. Although preliminary, this work provides a strong conceptual and methodological foundation for advancing interpersonally focused digital interventions for PPD and offers a framework for future innovation in perinatal mental health care.

## Conclusions

Despite the availability of effective treatments, up to 80% of women with PPD do not receive appropriate care^3,5,6^ resulting in significant and enduring gaps in maternal mental health services. Although pharmacologic and psychotherapeutic approaches can reduce depressive symptoms, they do not consistently address the relational disturbances and disruptions in mother–infant interaction that are central to PPD and associated with adverse child outcomes.^22^ Multiple structural and psychosocial barriers—further restrict the feasibility and accessibility of traditional in-person care. Effective interventions must therefore address both symptom reduction and dyadic functioning while overcoming barriers that impede equitable access to evidence-based treatment.^7,14^

To respond to these gaps, our team translated the MBIPT model used in our in-person dyadic program into a 12-session digitally delivered platform designed for AA/B women with integrated clinician- and patient-facing functionality. *MommaConnect* supports culturally tailored structured psychotherapy delivery and extends treatment between sessions through a psychoeducational library, mood monitoring, dyadic skill-building exercises, and secure clinician and patient messaging. This digitally enabled adaptation augments conventional in-person or telehealth psychotherapy by providing continuity of care and directly addressing both maternal depressive symptoms and mother–infant interaction difficulties.

Early development work has focused on formative research, usability evaluation, and iterative refinement with mothers and clinicians. Future phases include feasibility testing, clinical validation, implementation at scale, and the development of robust monitoring systems for safety, ethics, and dyadic–process analytics. This work has the potential to transform access to perinatal mental health care, improve maternal and infant outcomes, and reduce the long-term sequelae associated with untreated PPD and disrupted early relational experiences. Next steps include usability/feasibility testing, efficacy testing, clinical validation, deployment, monitoring ethics and safety, and further developing *MommaConnect* for the wider perinatal population. Broader implications include guiding policy reforms that incentivize dyadic perinatal care, informing clinical practice standards for digitally supported psychotherapy, and advancing the science of digital therapeutics tailored for maternal and infant mental health.

## Figures and Tables

**Table 1:** Process for Developing *MommaConnect*

Stage	Description	Methods Used
1	Understanding needs	Focus groups (mothers), In-depth Interviews (clinicians)
2	Translating therapy	Component mapping, expert panels
3	Designing the platform	Co-design, Prototyping, User-interface design methods
4	Testing usability	Think-aloud, Beta testing, heuristic evaluation (evaluators independently review the interface and identify usability issues).
5	Testing Feasibility& Efficacy	Feasibility and pilot studies
6	Clinical validation	RCTs, non-inferiority trials
7	Deploying	Implementation science methods
8	Monitoring development	Analytics, Ecological Momentary Assessment
9	Attending to Ethics & Safety	Privacy review, risk protocols

Note: This staged process is informed by established frameworks for perinatal mental health intervention development, digital psychotherapy translation, usability science, clinical trials methodology, and maternal–child health implementation.^32,38–49^
